# Executive Functions, Time Organization and Quality of Life among Adults with Learning Disabilities

**DOI:** 10.1371/journal.pone.0166939

**Published:** 2016-12-13

**Authors:** Kineret Sharfi, Sara Rosenblum

**Affiliations:** The Laboratory of Complex Human Activity and Participation, Department of Occupational Therapy, Haifa University, Haifa, Israel; Wuhan University, CHINA

## Abstract

**Purpose:**

This study compared the executive functions, organization in time and perceived quality of life (QoL) of 55 adults with learning disabilities (LD) with those of 55 matched controls (mean age 30 years). Furthermore, relationships and predictive relationships between these variables among the group with LD were examined.

**Methods:**

All participants completed the *Behavioral Rating Inventory of Executive Functions* (BRIEF-A), the *Time Organization and Participation* (TOPS, A-C) and the *World Health Organization Quality of Life* (WHOQOL) *questionnaires*. Chi-square tests, independent t-tests and MANOVA were used to examine group differences in each of the subscales scores and ratings of each instrument. Pearson correlations and regression predictive models were used to examine the relationships between the variables in the group with LD.

**Results:**

Adults with LD had significantly poorer executive functions (BRIEF-A), deficient organization in time abilities (TOPS A-B), accompanied with negative emotional response (TOPS- C), and lower perceived QoL (physical, psychological, social and environmental) in comparison to adults without LD. Regression analysis revealed that Initiation (BRIEF-A) significantly predicted approximately 15% of the participants' organization in time abilities (TOPS A, B scores) beyond group membership. Furthermore, initiation, emotional control (BRIEF-A subscales) and emotional responses following unsuccessful organization of time (TOPS-C) together accounted for 39% of the variance of psychological QoL beyond the contribution of group membership.

**Conclusions:**

Deficits in initiation and emotional executive functions as well as organization in time abilities and emotional responses to impairments in organizing time affect the QoL of adults with LD and thus should be considered in further research as well as in clinical applications.

## Introduction

Learning disabilities (LD) refer to a large group of neurological disorders caused by deficits in the central nervous system that influence the individual’s ability to efficiently maintain, process or convey information to others (e.g. [1]). The most prevalent current definitions of LD focus on impairments in academic skills [2], such as an imperfect ability to listen, think, speak, write, spell, or perform mathematical calculations [3]. Consequently, LD is usually diagnosed within educational systems (e.g. [2,4]). However, the literature reveals that children, as well as adolescents with LD may deal with social and emotional difficulties in addition to their academic difficulties (e.g. [5,6]). Studies on children, adolescents and adults with LD indicate frequent comorbidity with other health conditions described in the Diagnostic and Statistical Manual of Mental Disorders (DSM) [7]. For example it was reported that a high percentage (25–50%) are also diagnosed with attention deficit hyperactivity disorder (ADHD) (e.g. [8]), and that between 40–50% [9] also suffer from depression and anxiety disorders (e.g. [10,11]). Therefore, the latest version of the *DSM* (5^th^ Ed.) [7] includes a suggestion to identify LD based on a clinical synthesis of developmental, medical, family, and educational assessments and reports [4].

**Fig 1:**
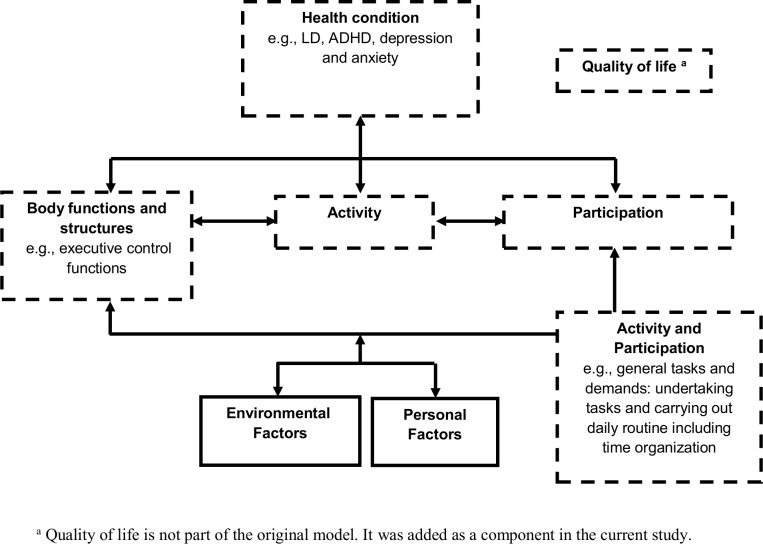
Components of the ICF model (WHO, 2001).

Given the described above, the design of this study employed the concepts of the International Classification of Functioning, Disability and Health (ICF) framework presented by the World Health Organization [12]. It has been suggested that this comprehensive health model is useful for the exploration of the needs of adults with LD in various life domains [13]. The central concepts of the ICF model include the following components: health condition, contextual factors, body functions and structures, activity and participation (Fig 1). *Health condition* refers to any disease, disorder or injury that exists in a person’s life, while *body functions and structures* include all human body parts and their functions. *Activity* describes the execution of specific tasks and *participation* describes involvement in a range of life situations [12]. Levels of activity and participation are viewed as outcomes of the interaction between health conditions and contextual factors.

### Executive functions

Executive functions (EF) are defined as the mental capacities involved in effective goal-directed activities (e.g. [14,15]). These functions are dependent on prefrontal brain areas (e.g. [16]) and on links between dorsolateral frontal and parietal neocortices (e.g. [17]). Current literature suggest that groups of brain regions cluster together into densely interconnected structures and that these interactions change during task execution (e.g. [18]). According to Elliott (2003), EF are involved in complex cognitions, such as solving novel problems, modifying behavior in light of new information, generating strategies, or sequencing complex actions [14]. The literature presents various definitions of the term of EF and of its constituent components [19]. According to the ICF model, EF are referred to as higher-level cognitive functions and are classified as a body function in the chapter that discusses mental functions [12]. In the current study, EFs are operationally defined according to the nine clinical scales in the Behavioral Rating Inventory of Executive Functions (BRIEF-A): inhibit; shift; emotional control; self-monitor; initiate; working memory; plan/organize; task monitor; and organization of materials [20]. These BRIEF-A scales relate to EF domains that include the ability to initiate behaviors, inhibit responses or competing actions, retain and manipulate information (working memory), select relevant task goals, plan and organize thoughts and behaviors, think flexibly and adapt to changes in one’s environment, regulate emotions, and monitor and evaluate one’s thoughts, emotions, and behaviors [19].

The literature reveals significant differences between children and adolescents with LD and controls with respect to a variety of EF components, while working memory, inhibition, initiation and set-shifting deficits were shown to be related to impaired abilities in reading, mathematics and writing [21,22,23,24,25]. In addition, research has demonstrated that *organizational abilities* significantly discriminated between children with and without dysgraphia [26]. However, the literature on EF among adults with LD is sparse. In her master's thesis Grinblat (2012) found that adult students with LD demonstrated significantly more difficulties in all BRIEF-A subscales and general scores compared to adult students without LD [27]. The findings of other studies have also indicated that children and adults with dyslexia have EF deficiencies relating to the ability to *inhibit* distractors and *sequence* events [28]. Moreover, Vasic and colleagues demonstrated functional differences in cortical regions associated with *language processing* and *EF* among adolescents and young adults with dyslexia [29], and Swanson found that *short term* and *working memory* were important in reading comprehension and mathematics performance in both children and adults with LD [30].

### Organization in time

The ICF relates to the concept of *time management* both as a component of Body Functions and also as a component of Activity and Participation, in the section on General Tasks and Demands. In the current study, time management was examined in the context of the participants' daily functioning and therefore will be considered as it relates to the component of Activity and Participation [12].

The organization of daily activities and their execution in time is an expression of how individuals proceed through the time cycle based on their personal temporal abilities [31]. These abilities are required for independent work and long-term projects [32]. Grinblat found that adult students with LD had significantly lower time perception ability and time organization performance as measured with the TOPS questionnaire compared with adult students without LD [27]. Efficacy and successful functioning under time constraints provide the individual with a sense of control, which is related to better physical health, academic achievement [33], and quality of life (QoL) [34]. Given the EF deficits found among individuals with LD and ADHD (e.g. [35,36]) and the impact of these deficits on the individual's quality of life (QoL) it seemed important to examine the perception of QoL within this population as well.

### Quality of Life

Quality of life (QoL) is a concept which broadly encompasses how individual persons measure the 'goodness' of multiple aspects of their lives [37]. The World Health Organization defines QoL as people's perception of their position in life in the context of the culture and value systems in which they live, and in relation to their goals, expectations, standards and concerns [38]. Most QoL conceptual models include a number of domains that focus on the following life dimensions: material, physical well-being, emotional well-being, social well-being and productivity [39]. This study employed the WHOQOL questionnaire to measure QoL in four domains: physical, psychological, social and environmental QoL [40]. The measure of QoL in the population of individuals with LD and co-occurring health conditions appears to be a new research interest, which has indicated low levels of QoL among children with LD [41] and among adults with ADHD [42].

### Aims of the study

The literature reveals that adults with LD may demonstrate EF deficits, which may be expressed as limitations in the temporal organization of their daily activity and thus influence their QoL. Therefore the aims of the present study were: (a) to compare executive functions, organization in time and perceived QoL among adults with LD and a matched control group; (b) to examine the relationships and predictive relationships between these variables in the group with LD using concepts from the ICF model.

## Methods

### Procedure

This study was designed as a quasi-experimental case-control design. It was approved by the ethics committee for human subject research of the Faculty of Social Welfare and Health Sciences at Haifa University. Data was collected between March 2011 and August 2012. The researcher met with each participant individually in a quiet location and obtained a signed written informed consent. Each participant completed a socio-demographic questionnaire followed by an extensive set of evaluations and questionnaires. Adults with LD were invited to have the questions read to them out loud, and to receive a free professional advisory hour in exchange for their participation.

The final sample size of 110 participants (55 adults with LD and 55 controls) was determined using a statistical power analysis program [43]. The G*Power3 program calculated a critical t of 1.659 with 108 df and a sample power of 0.831 for 21 central constructs which were examined with an effect size of 0.5 and α error probability of 0.05.

The inclusion criteria were: 20–50 years of age, mother tongue level of Hebrew reading and writing, intact vision and hearing or corrected with an aid, without motor or neurological disabilities, generally healthy with no chronic diseases or significant injuries that could influence daily activities and QoL. Participants in the study group were required to present proof of their LD diagnoses signed by a formal professional. With respect to the control group, only participants who could answer "no" to the following two questions were included: "Has anyone ever told you that you may have a LD?" and "Did you ever think you may have a LD?"

### Participants

A convenience sample of 110 adults from the Southern and Central regions of Israel participated in the study. Participants were invited to participate in the study via e-mails and Facebook. Those who wanted to participate in the study contacted the researcher and were asked initial questions to confirm inclusion criteria. Fifty-five adults presented proof of a formal diagnosis of LD and 55 adults were assigned to the control group. The two groups were matched for the following socio-demographic variables: gender, age, level of education and socio-economic status (α > 0.05).

### Instruments

Socio-demographic questionnaire. This self-report questionnaire includes 36 questions and was constructed for this study. Twenty-five questions relate to participants' socio-demographic status and the remaining 11 relate to their developmental background, their past experiences in high-school and to their employment history.

Behavioral Rating Inventory of Executive Functions–Adolescents/Adults version (BRIEF-A) [20] (Hebrew version). A 75- item self-report questionnaire that examines the behavioral manifestations of the examinee's EF. For each item, participants indicate how often they behave as described in the item on a 3-point scale. Higher grades indicate more difficulties in EF. Nine subscales are calculated: inhibition; set-shifting; emotional control; self-monitoring; task initiation; working memory; planning/organization; task monitoring; and organization of materials. These subscales are standardized to produce T-scores according to age and gender norms. In addition, two index scores are calculated: a behavioral regulation index (BRI) and a meta-cognition index (MI), as well as a global score referred to as the executive composite (GEC) score [20]. The BRIEF-A is known for its ecological validity [44] and for the similarity between its test items and daily, real-life challenges [45,46]. A validated translation to Hebrew was implemented in the current study. Initial results supported the internal consistency, structure validity and discriminant validity of the Hebrew version among adults with ADHD [47].

Time Organization and Participation (TOPS) [32]. A 35-item self-report questionnaire examines the subject's difficulties in time organization while performing daily tasks and serves as a functional measure of the subject's EF. The questionnaire comprises three parts and is rated on a 5-point rating scale. Part A requires the respondents to rate the extent to which they feel that they perform each daily activity at an appropriate *pace*, as expected in their the environment; in part B respondents rate their *performance* in time organization over the course of the day or within a certain period of time; in part C respondents rate the frequency of different *emotional responses* following unsuccessful organization of time. Two additional items in part D relate to the influence of change in routines and various stimuli on the individual’s organization of time abilities, but these items are used for clinical purposes [32] and were therefore not statistically analyzed for this study. Lower scores indicate higher risk of difficulties in organization and participation in time in daily tasks. High internal consistency was reported for the TOPS general score (α = 0.92) and for factors A, B and C (0.87 < α < 0.92) and construct validity was reported by differences in age groups [32].

World Health Organization Quality Of Life questionnaire (WHOQOL) [40]. A 26-item self-report questionnaire to measure the subject's self-perception of his/her QoL according to a 5-point rating scale. Participants rate their level of satisfaction or the level of accuracy of the items with respect to their lives over the past two weeks prior to completing the questionnaire. Scores for four domains are calculated: physical, psychological, social and environmental QoL. In this questionnaire higher grades indicate higher self-perception of QoL in each domain. Psychometric measures of this questionnaire are based on data from 23 different countries (N = 11,830) and include good to excellent internal reliability, inter-items correlations, discriminant validity and construct validity (based on factor analysis) [48]. In Israel, the Hebrew version was reported to have a good internal reliability in the unpublished master's thesis of Goldman (2010).

### Statistical analysis

Descriptive statistics were calculated for socio-demographic characteristics. Chi-square tests, independent t-tests and multiple analyses of variance (MANOVA) were used to examine between-groups differences in each of the subscales scores and ratings of each instrument. Pearson correlations and regression predictive models were used to examine the relationships between the variables.

## Results

### Socio-demographic characteristics

A detailed description of the sociodemographic characteristics of the participants was reported previously, with no significant differences found between the groups' age, gender, socio-economic status and level of education [49].

The group of adults with LD (n = 55) included 34.5% males and 64.5% females ages 20–46 years with mean age 29.58 (SD = 6.4) and the control group (n = 55) included 23.6% males and 76.4% females ages 23–47 with mean age 31.18 (SD = 6.4). This gender ratio is a result of the voluntary turns of the participants and does not represent the gender ratio reported in previous literature (e.g. [50]).

### Differences between adults with and without LD in executive functions, organization in time and perceived quality of life

#### Executive functions

A MANOVA across all nine subscales of the BRIEF-A yielded statistically significant differences between the two groups (F [1,108] = 14.47, Wilks' Lambda = 0.43, p < .001, η^2^ = 0.57). As shown in Table 1, the subsequent univariate ANOVA analyses revealed statistically significant differences for the mean scores obtained in all nine subscales (p < .005) with an effect size of 0.57. The largest/maximal difference of all the subscale scores was in the working memory score. In addition, independent t-tests yielded significant differences between the two groups for the behavioral regulation index (BRI) (t[108] = -6.05, p < .001), the meta-cognition index (MI) (t[108] = -8.78, p < .001) and the global executive composite (GEC) score (t[108] = -7.30, p < .001).

**Table 1:** Means and standard deviations of *BRIEF-A*^a^ subscale scores, indexes and GEC^b^ score.

BRIEF-A subscales, Indexes and GEC score	Adults with LD^c^ Mean (SD)	Controls Mean (SD)	F -Value	η^2^
**Inhibition**	59.73 (10.54)	47.93 (8.05)	43.510**	.29
**Set-shifting**	61.69 (9.96)	52.33 (9.20)	26.230**	.19
**Emotional control**	59.16 (10.64)	52.78 (9.20)	10.494*	.09
**Self-monitoring**	55.11 (10.66)	47.33 (7.89)	18.945**	.15
**Task initiation**	60.51 (10.29)	48.64 (8.20)	44.783**	.29
**Working memory**	70.89 (11.55)	49.64 (7.27)	133.423**	.55
**Planning/organization**	62.89 (11.80)	50.53 (8.31)	40.351**	.27
**Task monitoring**	64.47 (10.81)	51.60 (8.34)	48.898**	.31
**Organization of materials**	58.40 (13.52)	48.87 (8.75)	19.251**	.15
			**T value**	
**BRI**^**d**^	60.96 (9.69)	50.55 (8.32)	-6.050**	
**MI**^**e**^	65.67 (11.19)	49.93 (7.20)	-8.777**	
**GEC**	64.53 (9.99)	50.78 (9.77)	-7.298**	

*p ≤ 0.05

**p ≤ 0.001

^a^BRIEF-A = Behavioral Rating Inventory of Executive Functions–Adolescents/Adults Version

^b^GEC = Global executive composite

^c^LD = Learning disabilities

^d^BRI = Behavioral regulation index

^e^MI = Meta-cognition index

#### Organization in time

A MANOVA across the TOPS subscales A, B, and C, yielded statistically significant differences between the two groups (F [1,108] = 17.90, Wiks' Lambda = 0.34 p < .001, η^2^ = 0.34). As shown in Table 2, the subsequent univariate ANOVA analyses revealed statistically significant differences between the mean scores of all three subscales (p < .001) with an effect size of 0.34.

**Table 2:** Means and standard deviations of *TOPS*^a^ subscale scores and general score.

TOPS subscales and general score	Adults with LD^b^ Mean (SD)	Controls Mean (SD)	F/t-Value	η^2^
**Factor A (pace)**	3.64 (0.66)	4.32 (4.96)	37.46*	.26
**Factor B (performance)**	3.11 (0.80)	3.92 (0.64)	34.97*	.24
**Factor C (emotional reactions)**	3.27 (0.64)	3.85 (0.54)	26.46*	.20

*p < .001

^a^TOPS = Time Organization and Participation

^b^LD = Learning disabilities

#### Quality of life

A MANOVA across the four subscales of the WHOQOL yielded statistically significant differences between the two groups (F [1,108] = 5.36, Wilk's Lambda = 0.17, p < .005, η^2^ = 0.17). As shown in Table 3, the subsequent univariate ANOVA analyses revealed statistically significant differences between the mean scores of all four domains scores (p < .05) with effect size of 0.17.

**Table 3:** Means and standard deviations of *WHOQOL*^a^ domain scores.

WHOQOL domains scores	Adults with LD^b^ Mean (SD)	Controls Mean (SD)	F-Value	η^2^
**Physical QoL**^**c**^	15.23 (2.19)	16.56 (2.01)	10.99**	.09
**Psychological QoL**	13.99 (2.22)	15.72 (2.02)	18.35***	.14
**Social QoL**	14.59 (3.12)	15.85 (2.69)	5.16*	.05
**Environmental QoL**	14.65 (1.93)	15.67 (1.80)	8.19*	.07

*p ≤ 0.05

**p ≤ 0.05

***p ≤ 0.001

^a^WHOQOL = World Health Organization Quality of Life questionnaire

^b^LD = Learning disabilities

^c^QoL = Quality of life

### Correlations between executive functions, organization in time and quality of life among adults with LD

Pearson correlations examined the relationships between executive functions, organization in time and perceived quality of life among adults with LD. As shown in Table 4 significant relationships were found between the executive functions of adults with LD and their organization in time abilities and QoL. In addition, the organization in time abilities of adults with LD were significantly correlated with their QoL scores.

**Table 4:** Correlations between subscale scores of the BRIEF-A, TOPS, and WHOQOL questionnaires (n = 55).

		TOPS^2^			WHOQOL^3^			
		A	B	C	Phy	Psy	Soc	Env
**BRIEF-A**^**1**^	Inhib	-.39**		-.40**		-.37**	-.31*	
	Se-S			-.46***		-.43**	-.41**	-.37**
	EmC	-.36**		-.53***		-.43**	-.40**	
	Init	-.43**	-.41**	-.49***	-.34*	-.57***	-.33*	-.45**
	WM	-.37**	-.39**		-.38**	-.38**		-.39**
	P/O	-.33*	-.38**		-.44**	-.39**		-.33*
	TM	-.43**	-.48***	-.35**		-.32*		-.32*
	OoM	-.40**	-.39**			-.51***	-.45**	
	BRI	-.39**		-.56***	-.38**	-.43**		-.41**
	MI	-.45**	-.49***	-.33**	-.38**	-.54***	-.38**	-.38**
	GEC	-.50***	-.46***	-.48***		-.37**	-.31*	
**TOPS**^**2**^	A					.36**		.42**
	B				.35**			.38**
	C					.55***	.38**	

Only significant correlations of r > 0.3 are presented in the table. Variables that did not significantly correlate were removed from the table.

* p < 0.05

** p < 0.01

*** p < 0.001

^1^*BRIEF-A* = *The Behavioral Rating Inventory of Executive Functions*; Inhib = Inhibition; Se-S = Set-shifting; EmC = Emotional control; Init = Task initiation; WM = Working memory; P/O = Planning/Organization; TM = Task monitoring; OoM = Organization of materials; BRI = Behavioral Regulation Index; MI = Meta-cognitive Index; GEC = General Executive Composite

^2^*TOPS* = Time Organization and Participation

^3^*WHOQOL* = The World Health Organization Quality Of Life questionnaire; Phy = Physical QoL; Psy = Psychological QoL; Soc = Social QoL; Env = Environmental QoL

### Executive Functions as predictors of organization in time abilities among adults with LD

Table 5 presents the prediction of pace and performance of organization in time (TOPS-A and TOPS-B respectively) and of the emotional responses following unsuccessful organization of time (TOPS-C) by the executive functions (BRIEF-A) subscales.The group accounted for 25.8% of the variance (F [1,108] = 37.46, p < .001) in prediction of TOPS-A, 24.5% of the variance (F [1,108] = 34.97, p < .001) in prediction of TOPS-B, and 19.7% of the variance (F [1,108] = 26.46, p < .001) in the prediction of the emotional responses following unsuccessful organization of time (TOPS-C).

**Table 5:** Predicting *TOPS*^a^ subscales scores from group membership and *BRIEF-A*^b^ subscales scores.

	TOPS-A			TOPS-B			TOPS-C		
Variable	B	SE B	β	B	SE B	β	B	SE B	β
**Group**	-.30	.12	-.22*	-.34	.14	-.20*	-.15	.10	-0.12
**Emotional control**							-.03	.00	-.51***
**Initiation**	-.02	.01	-.35***	-.03	.01	-.37***	-.02	.00	-.33***
**Organization of materials**	-.01	.01	-.24**	-.01	.01	-.22*			
Adj R^2^ **=**	0.43			0.42			0.57		
*F* **=**	28.29***			27.36***			48.31***		

*p ≤ 0.05

**p ≤ 0.01

***p ≤ 0.001

^a^ TOPS = Time Organization and Participation

^b^ BRIEF-A = Behavioral Rating Inventory of Executive Functions–Adolescents/Adults Version

As presented in Table 5, initiation (BRIEF A) accounted for all the three TOPS scores (A-C) while the organization of materials subscale (BRIEF–A) accounted for both TOPS-A and TOPS-B scores and emotional control for TOPS-C score, beyond the contribution of group membership.

#### TOPS-A: pace of organization in time

Initiation accounted for 14.5% (F [1,107] = 26.05, p < .001) and organization of materials accounted for 4.2% of the variance (F [1,106] = 7.97, p < .01). As a whole, those two EF subscales accounted for 18.7% of the variance of the TOPS-A score beyond the contribution of group membership.

#### TOPS-B: Performance of organization in time

Initiation (BRIEF-A) accounted for 15.5% (F [1,107] = 27.67, p < .001) and organization of materials (BRIEF-A) accounted for 3.7% of the variance (F [1,106] = 6.87, p < .05). As a whole, those two EF subscales accounted for 19.1% of the variance of the TOPS-B score, beyond the contribution of group membership.

#### TOPS-C: Emotional responses following unsuccessful organization of time

Emotional control (BRIEF-A) accounted for 31.1% (F [1,107] = 67.75, p < .001) and initiation (BRIEF-A) accounted for 6.9% of the variance (F [1,106] = 17.41, p < .001). As a whole, those two EF subscales accounted for 38.1% of the variance of the TOPS-C score beyond the contribution of group membership.

### Executive functions and organization in time abilities as predictors of quality of life

Table 6 presents the prediction of psychological QoL (WHOQOL). The group accounted for 14.5% of the variance in psychological QoL (F [1,108] = 18.35, p < .001). Initiation (BRIEF-A) accounted for 24% (F [1,107] = 41.77, p < .001), emotional control (BRIEF-A) accounted for 11.6% (F [1,106] = 24.62, p < .001), and TOPS-C subscale score added 4.3% of the variance to the prediction of perceived psychological QOL (F [1,105] = 9.92, p < .01) beyond the contribution of group membership. As a whole, those two EF subscales together with the emotional responses following unsuccessful organization of time accounted for 39.9% of the variance of the psychological QoL score, beyond the contribution of group membership.

**Table 6:** Predicting psychological QoL^a^ domain (*WHOQOL*)^b^ from group membership, *BRIEF-A*^c^ subscales scores and *TOPS-C*^*d*^ subscale score.

Psychological QOL			
Variable	B	SE B	β
Group	.09	.36	.02
Initiation	-.08	.02	-.36***
Emotional control	-.04	.02	-.21*
TOPS-C	1.10	.35	.32**
Adj R^2^ =	0.53		
*F* =	31.34***		

*p ≤ 0.05

**p ≤ 0.01

***p ≤ 0.001

^a^QoL = Quality of life

^b^WHOQOL = *World Health Organization Quality of Life questionnaire*

^c^BRIEF-A = Behavioral Rating Inventory of Executive Functions–Adolescents/Adults Version

^d^TOPS-C = *Time Organization and Participation*

## Discussion

The purpose of this study was to: (a) compare executive functions, organization in time and perceived QoL between adults with LD and a matched control group; and (b) examine the relationships and predictive relationships between these variables among the group with LD using concepts from the ICF model.

In this study differences between the groups were found in all the variables that were examined. However, gender ratios in this sample were not representative of previously reported gender ratios among the population with LD (e.g. [50]). Therefore the results should be interpreted cautiously.

Results indicated that adults with LD had significantly more executive functions deficiencies than a matched control group. This finding strengthens the initial results of Grinblat regarding EF deficiencies among adult students with LD [27], and is in line with previous reports on relationships between deficiencies in EF and LD among children and adolescents of various ages ([21,22,23,24,25,26]). Such relationships are in line with findings about the association of EF with school readiness (e.g. [51,52]), school success (e.g. [53,54,55] and job success (e.g. [56]) and raise questions as to the role of executive functions deficiencies in the academic, social and emotional performance of this population.

The largest/maximal difference between the groups in their executive functions was in their working memory score. Adults with LD had significantly more difficulties with working memory compared to their matched controls. This finding is in line with previous literature regarding working memory deficits among children and adolescents with LD (e.g. [21,22,25]) and testifies as to the continuing nature of LD into adulthood.

The finding that adults with LD had more limitations in the pace and performance of time organization and more emotional responses following unsuccessful organization of time compared to their matched controls is in line with previous findings [27]. In fact, organization in time is considered to be based upon executive function processes [32] and is required for successful participation in other domains of daily life besides the academic domain, such as home, work, social participation and so on. Thus, further investigation is required with larger samples focusing on daily activities performance characteristics of adults with LD in various life domains beside the academic domain [13].

In the current study adults with LD perceived their QoL to be lower than that of their peers without LD. This strengthens a previous report of lower QoL found among children with LD [41]. Such findings demonstrate that the health picture of this population is a complicated one and that the lives of these individuals are affected in a comprehensive manner that includes their physical, psychological, social and environmental QoL and not only their academic performance. Lower QoL was reported previously among adults with ADHD [42] and among adults with depression [57]. Further investigation of possible relationships between those health conditions and LD and their dynamic influence on the QoL of adults with LD is recommended.

In this study organization in time of adults with LD was significantly predicted by certain aspects of their executive functions. For example, initiation significantly predicted approximately 15% of the pace and performance of organization in time beyond the contribution of group membership. As far as we know, this is the first study of its kind in which these variables were examined among individuals with LD in general and among adults with LD specifically. Further investigation is recommended in order to achieve a better understanding of the relationships between the deficits of adults with LD in working memory and other EFs and their difficulties in the initiation of tasks. Difficulties with working memory were reported in the past in relation to daily functioning (e.g. [58]). The results of this study indicate that deficits in the initiation of tasks may underlie these difficulties in daily functioning. Furthermore, future studies should explore other variables which might be associated with the difficulties of adults with LD in the initiation of tasks, such as differences in their sensory profiles [50].

Another unique and yet plausible finding is that executive emotional control was a significant predictor of the emotional reactions to unsuccessful organization in time beyond the contribution of group membership. In a study by Barkley and Murphy in 2011, they argued that EF self-report and other-reported ratings are more strongly associated than scores on EF tests with impairments in major life activities among adults with ADHD [59]. However both the BRIEF-A and the TOPS which were used in this study examine the functional expressions and behavioral manifestations of EF. Therefore additional tests should be administered in order to neutralize the possibility that the examined emotional constructs measured in the two instruments used in this study were too similar, in order to further validate these results.

The results that indicated that the EF of emotional control and initiation as well as the emotional reactions to unsuccessful organization in time were significant predictors of the psychological QoL of adults with LD beyond the contribution of group membership add further insights into this population. Impaired EF has been reported in the past as related to low levels of QoL (e.g. [37,38]). However this finding suggests that the lower psychological QoL of adults with LD may be related not only to their primary deficits in EF but also to secondary emotional reactions to their difficulties in everyday task functions including time organization. These may have a boomerang effect on the executive functions abilities of the person with LD, for example by developing depression, a condition that is commonly found in this population [10,11]. This in turn may be expressed as occupational and social impairments (e.g. [60]) as well as a lower QoL (e.g. [57]) as previously described to exist among adults with depressive disorders.

In accordance with the ICF model concepts, the results of this study demonstrate that adults with LD confront deficiencies in body functions and that these deficiencies are expressed as limitations in their activity and participation in a variety of life domains and not only in their academic performance. These findings strengthen the need to consider this population within a wide health perspective and not only in terms of their academic deficiencies for which they are usually diagnosed and treated [2,4]. In addition, such deficiencies and limitations need to be considered in the clinical evaluation process of adults with LD in order to provide better and more effective intervention programs. In the original ICF model, the relationships between the ICF components and QoL were addressed as an issue that requires further examination [12]. However, a modified ICF model version was proposed lately by McDougall and colleagues [61]. In this modified version it is thought that all of the ICF model components could potentially affect a person’s QoL and contribute to changes in QoL as the person develops over time [62]. Therefore the finding in this study regarding lower perceived QoL among adults with LD indicates that this population should be considered a population at risk and should be treated with preventative and intervention programs in order to avoid the development of secondary emotional complications and decreased QoL.

## Abbreviations

QoL: Quality of life
LD: Learning disabilities
BRIEF-A: Behavioral Rating Inventory of Executive Functions–Adult version
TOPS: Time Organization and Participation Scale
WHOQOL: World Health Organization Quality of Life questionnaire
DSM: Diagnostic and Statistical Manual of Mental Disorders
ADHD: Attention Deficit Hyperactivity Disorder
ICF: International classification of Functioning, Disability and Health
EF: Executive Functions
MANOVA: multiple analyses of variance.

## References

[1] Kavale KA, Forness SR. What definitions of learning disabilities say and don't say–A critical analysis. J Learn Disabil. 2000;33: 239–256. 15505963 10.1177/002221940003300303

[2] Scanlon D. Specific learning disability and its newest definition: Which is comprehensive and which is insufficient? J Learn Disabil. 2013;46: 26–33. 10.1177/0022219412464342 23144061

[3] Kavale KA, Spaulding LS, Beam AP. A time to define: Making the specific learning disability definition prescribe specific learning disability. Learn Disabil Q. 2009;32: 39–48.

[4] Cavendish W. Identification of learning disabilities: Implications of proposed DSM-5 criteria for school-based assessment. J Learn Disabil. 2013;46: 52–57. 10.1177/0022219412464352 23128455

[5] Bryan T, Burstein K, Ergul C. The social-emotional side of learning disabilities: A science-based presentation of the state of the art. Learn Disabil Q. 2004;27: 45–51.

[6] Kavale KA, Forness SR. Social skill deficits and learning disabilities: A meta-analysis. J Learn Disabil. 1996;29: 226–237. 8732884 10.1177/002221949602900301

[7] American Psychiatric Association. Diagnostic and Statistical Manual of Mental Disorders: DSM-5. Washington, D.C.; American Psychiatric Association 2013; Available: http://www.psychiatry.org/File%20Library/Practice/DSM/DSM-5/DSM-5-TOC.pdf

[8] Barkley RA. What to look for in a school for a child with ADHD. ADHD Report. 1994;2: 1–3.

[9] Greenhill LL. Learning disabilities: Implications for psychiatric treatment Washington, DC: American Psychiatric Press; 2000.

[10] Peleg O. Test anxiety, academic achievement, and self-esteem among Arab adolescents with and without learning disabilities. Learn Disabil Q. 2009;32: 11–20.

[11] Wilson AM, Armstrong CD, Furrie A, Walcot E. The mental health of Canadians with self-reported learning disabilities. J Learn Disabil. 2009;42: 24–40. 10.1177/0022219408326216 19103798

[12] World Health Organization. International Classification of Functioning, Disability and Health. Geneva: Author; 2001.

[13] Sharfi K, Rosenblum S. Activity and participation characteristics of adults with learning disabilities: A systematic review. PLoS One. 2014;9: 1–8. 10.1371/journal.pone.0106657 PMC4153678 25184315

[14] Katz N, Maeir A. Higher level cognitive functions enabling participation: Awareness and executive functions In Katz N, editor. Cognition, occupation, and participation across the life span. Bethesda, MD: The American Occupation Association; 2011 pp. 13–40.

[15] Lezak MD, Howieson DB, Loring DW, Hannay HJ, Fischer JS. Neuropsychological assessment 4th ed. New York: Oxford University Press; 2004.

[16] Funahashi S. Neuronal mechanisms of executive control by the prefrontal cortex. Neurosci Res. 2001;39: 147–165. 11223461 10.1016/s0168-0102(00)00224-8

[17] Seeley WW, Menon V, Schatzberg AF, Keller J, Glover GH, Kenna H,. . . Greicius MD. Dissociable intrinsic connectivity networks for salience processing and executive control. J Neurosci. 2007;27: 2349–2356. 10.1523/JNEUROSCI.5587-06.2007 17329432 PMC2680293

[18] Braun U, Schäfer A, Walter H, Erk S, Romanczuk-Seiferth N, Haddad L,… Meyer-Lindenberg A. Dynamic reconfiguration of frontal brain networks during executive cognition in humans. Proc Natl Acad Sci U S A. 2015;112: 11678–11683. 10.1073/pnas.1422487112 26324898 PMC4577153

[19] Roth RM, Lance CE, Isquith PK, Fischer AS, Giancola PR. Confirmatory factor analysis of the Behavior Rating Inventory of Executive Function-Adult version in healthy adults and application to Attention-Deficit/Hyperactivity Disorder. Arch Clin Neuropsychol. 2013;28: 425–434. 10.1093/arclin/act031 23676185 PMC3711374

[20] Roth RM, Isquith PK, Gioia GA. Behavior Rating Inventory of Executive Function—Adult version (BRIEF-A). Lutz, FL: Psychological Assessment Resources; 2005.

[21] Bull R, Scerif G. Executive functioning as a predictor of children’s mathematics ability: Inhibition, switching, and working memory. Dev Neuropsychol. 2001;19: 273–293. 10.1207/S15326942DN1903_3 11758669

[22] Christopher ME, Miyake A, Keenan JM, Pennington B, DeFries JC, Wadsworth SJ, Willcutt E. . . . Olson RK. Predicting word reading and comprehension with executive function and speed measures across development: A latent variable analysis. J Exp Psychol Gen. 2012;141: 470–488. 10.1037/a0027375 22352396 PMC3360115

[23] Gioia GA, Isquith PK, Kenworthy L, Barton RM. Profiles of everyday executive function in acquired and developmental disorders. Child Neuropsychol. 2002;8: 121–137. 10.1076/chin.8.2.121.8727 12638065

[24] Hooper SR, Swartz CW, Wakely MB, de Kruif REL, Montgomery JW. Executive functions in elementary school children with and without problems in written expression. J Learn Disabil. 2002;35: 57–68. 15490900 10.1177/002221940203500105

[25] Toll SWM, Van der Ven SHG, Kroesbergen EH, Van Luit JEH. Executive functions as predictors of math learning disabilities. J Learn Disabil. 2011;44: 521–532. 10.1177/0022219410387302 21177978

[26] Rosenblum S, Aloni T, Josman N. Relationships between handwriting performance and organizational abilities among children with and without dysgraphia: A preliminary study. Res Dev Disabil. 2010;31: 502–509. 10.1016/j.ridd.2009.10.016 19945252

[27] Grinblat N, Rosenblum S. Time perception, executive functions, temporal organization and participation among students with and without learning disabilities. M. Sc. University of Haifa, Israel. 2012. Available: http://digitool.haifa.ac.il/view/action/singleViewer.do?dvs=1459257046033~562&locale=iw_IL&VIEWER_URL=/view/action/singleViewer.do?&DELIVERY_RULE_ID=3&adjacency=N&application=DIGITOOL-3&frameId=1&usePid1=true&usePid2=true

[28] Brosnan M, Demetre J, Hamill S, Robson K, Shepherd H, Cody G. Executive functioning in adults and children with developmental dyslexia. Neuropsychologia. 2002;40: 2144–2155. 12208010 10.1016/s0028-3932(02)00046-5

[29] Vasic N, Lohr C, Steinbrink C, Martin C, Wolf RC. Neural correlates of working memory performance in adolescents and young adults with dyslexia. Neuropsychologia. 2008;46: 640–648. 10.1016/j.neuropsychologia.2007.09.002 17950764

[30] Swanson HL. Short-term memory and working memory: Do both contribute to our understanding of academic achievement in children and adults with learning disabilities? J Learn Disabil. 1994;27: 34–50. 8133185 10.1177/002221949402700107

[31] Rosenblum S, Regev N. Timing abilities among children with developmental coordination disorders (DCD) in comparison to children with typical development. Res Dev Disabil. 2013;34: 218–227. 10.1016/j.ridd.2012.07.011 22960066

[32] Rosenblum S. Validity and reliability of the Time Organization and Participation Scale (TOPS). Neuropsychol Rehabil. 2012;22: 65–84. 10.1080/09602011.2011.640465 22264145

[33] Lachman ME, Burack OR. Planning and control processes across the life span: An overview. Int J Behav Dev. 1993;16: 131–143.

[34] Farnworth L, Fossey E. Occupational terminology interactive dialogue. Explaining the concepts of time use, tempo and temporality. J Occup Sci. 2003;10: 150–153.

[35] Brown TE, Landgraf JM. Improvements in executive function correlate with enhanced performance and functioning and health-related quality of life: Evidence from two large, double-blind, randomized, placebo-controlled trials in ADHD. Postgrad Med. 2010;122: 42–51. 10.3810/pgm.2010.09.2200 20861587

[36] Stern A, Pollak Y, Bonne O, Malik E, Maeir A. The relationships between executive functions and quality of life in adults with ADHD. J Atten Disord. 2013; 1–8. 10.1177/1087054713504133 24189201

[37] Theofilou P. Quality of life: Definition and measurement. Eur J Psychol. 2013;9: 150–162.

[38] WHOQOL Group. Development of the WHOQOL: Rationale and current status. Int J Ment Health. 1994;23: 24–56.

[39] Felce D, Perry J. Quality of life: The scope of the term and its breadth of measurement In Brown RI, Editor. Quality of life for people with disabilities. 2nd ed London: Stanley Thornes Publishers 1997 pp. 56–71.

[40] World Health Organization (WHO). The World Health Organization Quality of Life Questionnaire–(WHOQOL)-BREF. Geneva: Author; 2004.

[41] Karande S, Bhosrekar K, Kulkarni M, Thakker A. Health-related quality of life of children with newly diagnosed specific learning disability. J Trop Pediatr. 2008;55: 160–169. 10.1093/tropej/fmn099 19042966

[42] Brod M, Johnston J, Able S, Swindle R. Validation of the Adult Attention-Deficit/Hyperactivity Disorder Quality-of-Life Scale (AAQoL): A disease-specific quality-of-life measure. Qual Life Res. 2006;15: 117–129. 10.1007/s11136-005-8325-z 16411036

[43] Faul F, Erdfelder E, Land AG, Buchner A. G*Power 3: A flexible statistical power analysis program for the social, behavioral, and biomedical sciences. Behav Res Methods. 2007;39: 175–191. 17695343 10.3758/bf03193146

[44] Vriezen ER, Pigott SE. The relationship between parental report on the BRIEF and performance based measures of executive function in children with moderate to severe traumatic brain injury. Child Neuropsychol. 2002;8: 296–303. 10.1076/chin.8.4.296.13505 12759826

[45] Koven NC, Thomas W. Mapping facets of alexithymia to executive dysfunction in daily life. Pers Individ Dif. 2010;49: 24–28.

[46] Taylor HG. Research on outcomes of pediatric traumatic brain injury: Current advances and future directions. Dev Neuropsychol. 2004;25: 199–225. 10.1080/87565641.2004.9651928 14984335

[47] Rotenberg-Shpigelman S, Rapaport R, Stern A, Hartman-Maeir A. Content validity and internal consistency reliability of the Behavior Rating Inventory of Executive Function-Adult version (BRIEF-A) in Israeli adults with Attention-Deficit/Hyperactivity Disorder. IJOT. 2008;17: H77–H96.

[48] Skevington SM, Lotfy M, O'connell KA. The World Health Organization’s WHOQOL-BREF quality of life assessment: Psychometric properties and results of the international field trial. A Report from the WHOQOL Group. Qual Life Res. 2004;13: 299–310. 10.1023/B:QURE.0000018486.91360.00 15085902

[49] Sharfi K, Rosenblum S. Sensory modulation and sleep quality among adults with learning disabilities: A quasi-experimental case-control design study. PLoS One. 2015;10: 1–14. 10.1371/journal.pone.0115518 PMC4319813 25658647

[50] Cortiella C, Horowitz SH. The State of Learning Disabilities. 3rd ed New York, NY: National Center for Learning Disabilities; 2014.

[51] Blair C, Razza RP. Relating effortful control, executive function, and false belief understanding to emerging math and literacy ability in kindergarten. Child Dev. 2007;78: 647–663. 10.1111/j.1467-8624.2007.01019.x 17381795

[52] Morrison FJ, Ponitz CC, McClelland MM. Self-regulation and academic achievement in the transition to school In: Calkins SD, Bell M, editors. Child development at the intersection of emotion and cognition. Washington, DC: American Psychological Association; 2010 pp. 203–224.

[53] Borella E, Carretti B, Pelegrina S. The specific role of inhibition in reading comprehension in good and poor comprehenders. J Learn Disabil. 2010;43: 541–552. 10.1177/0022219410371676 20606207

[54] Duncan GJ, Dowsett CJ, Claessens A, Magnuson K, Huston AC, Klebanov P, et al School readiness and later achievement. Dev Psychol. 2007;43: 1428–1446. 10.1037/0012-1649.43.6.1428 18020822

[55] Gathercole SE, Pickering SJ, Knight C, Stegmann Z. Working memory skills and educational attainment: Evidence from national curriculum assessments at 7 and 14 years of age. Appl Cogn Psychol. 2004;18: 1–16.

[56] Bailey CE. Cognitive accuracy and intelligent executive function in the brain and in business. Ann N Y Acad Sci. 2007;1118: 122–141. 10.1196/annals.1412.011 17717092

[57] Pyne JM, Patterson TL, Kaplan RM, Ho S. Preliminary longitudinal assessment of quality of life in patients with major depression. Psychopharmacol Bull. 1997;33: 23–29.‏ 9133748

[58] Kane MJ, Brown LH, McVay JC, Silvia PJ, Myin-Germeys I, Kwapil TR. For whom the mind wanders, and when: An experience-sampling study of working memory and executive control in daily life. Psychol Sci. 2007;18: 614–621. 10.1111/j.1467-9280.2007.01948.x 17614870

[59] Barkley RA, Murphy KR. The nature of executive function (EF) deficits in daily life activities in adults with ADHD and their relationship to performance on EF tests. J Psychopathol Behav Assess. 2011;2: 137–158.

[60] Godard J, Grondin S, Baruch P, Lafleur MF. Psychosocial and neurocognitive profiles in depressed patients with major depressive disorder and bipolar disorder. Psychiatry Res. 2011;190: 244–252. 10.1016/j.psychres.2011.06.014 21764461

[61] McDougall J, Wright V, Rosenbaum P. The ICF model of functioning and disability: Incorporating quality of life and human development. Dev Neurorehabil. 2010;13: 204–211. 10.3109/17518421003620525 20450470

[62] McDougall J, Wright V, Schmidt J, Miller L, Lowry K. Applying the ICF framework to study changes in quality-of-life for youth with chronic conditions. Dev Neurorehabil. 2011;14: 41–53. 10.3109/17518423.2010.521795 21034288 PMC4245180

